# Medical Opinion and Movement

**Published:** 1906-07-07

**Authors:** 


### Medical Opinion and Movement.

The appointments on the consulting medical staff and to the Executive Committee of King Edward VII. Sanatorium, as approved by His Majesty, must increase public confidence, and prove highly satisfactory to the profession and to the public. The names will be found on another page of the present issue. It is a memorable fact that the Advisory Committee has been strengthened by the addition of such names as Professor Clifford Albutt, Professor Osler, Dr. Goodhart, and Dr. Kingston Fowler. The Executive Committee, too, is a strong one, and affords the best guarantee that the administration of the sanatorium will be conducted on business-like and intelligent principles.

Much dissatisfaction has been caused by the action taken by the British Medical Association in regard to the election of direct representatives upon the Medical Council. The draft charter does not confer any power upon the Association to take part in these elections directly, and it is significant as showing the trend of medical opinion that the Council should have unanimously deleted from Clause 2 (2) (V) the power to promote the candidature of any member of the Association for Parliament or for any legislative assembly. Having thus unanimously resolved to keep the British Medical Association free from politics, it may be trusted, we hope, to set its face against any attempt to degrade the direct representatives on the General Medical Council to the level of mere delegates of one Association, however large it may be.

The discussion which has taken place in the columns of the *British Medical Journal* on the subject of the recognition of medical training schools in connection with the workhouse infirmaries has convinced most impartial members of the profession that the Central Midwives Board will have to amend its policy or it will grievously fail to do its duty. It is a remarkable fact that on the list of training schools for midwives, recognised by the Board, there is not a single Union infirmary licensed in London and only seven outside London. At the last meeting of the Midwives Board a licence was refused to the Whitechapel Union Infirmary, despite a letter from the Clerk of the London County Council and a suggestion that the matter be referred to the Standing Committee. We hope medical members of the Midwives Board will voice public and official opinion by getting the question of the recognition of the licensing of poor-law maternity departments referred to the Standing Committee, with instructions to inquire and report fully upon the whole question. The extraordinary views which Sir William Sinclair has enunciated in his letter of June 13th created no little astonishment in many quarters. They would indeed be amusing if the questions at issue were not so grave from a public and professional point of view.

The Council of the British Medical Association at their last meeting had under consideration the draft charter, and made certain material alterations in it. Broadly, the effect of these changes was, so far as they were material, to diminish the powers of the representative meeting and to increase those of the Council. As amended, the draft charter now vests the whole of the Association's affairs, funds, income and expenditure in the Council, and the representative meeting will merely have the general control and direction of the policy of the Association. It is not easy in any circumstances to organise a great association extending throughout the Empire, and we incline to the feeling that on the whole the Westminster division of the Metropolitan Counties branch have justification for resolving, with one dissentient, that the Royal charter would be detrimental to the best interests of the British Medical Association. In any case, much interest has been excited by Doctor Helme's notice of motion for the Council meeting on Wednesday, in which he seeks to pledge the Council to the opinion that further and fuller consideration of the whole question of a Royal charter is desirable, and that the representative meeting should be recommended to delay the application to the Privy Council until the Association as a whole has had longer time to appreciate and consider the many important matters involved.

In a speech upon sanatoria for tuberculosis Mr. Chamberlain expressed the opinion that the most efficient way for millionaires to spend their money would be to endow medical research, and this speech was quickly followed by the announcement that the King had been pleased to confer upon Dr. A. E. Wright the honour of knighthood. This is the first occasion in England upon which a medical man has obtained public recognition whose life's work has been concerned in the investigation of disease rather than in the practice of his profession. In the opinion of many no man's life is successful unless his energies have rewarded him with at least his fair share of money. When, however, a medical scientist starts on his life's career he knows that, although he may be possessed of the very highest abilities and the most untiring energy, and that although he may succeed in adding much to knowledge of lasting benefit to mankind, should he not be possessed of private means, he may have to subsist on a mere pittance, receiving, it may be, no more for a year's work than a surgeon charges for one operation. We are willing to pay well for anything that comes directly under our notice, especially, perhaps, when it obviously benefits ourselves; but to give money for what appear to be hazy probabilities is considered unpractical. There are pleasures, however, in investigating the unknown, and a satisfaction in feeling that work accomplished has helped to lift medical knowledge a step onward, which those who think much of money cannot appreciate. Of such pleasure and satisfaction Dr. Wright has experienced much, yet we do not doubt that he will feel not only the deepest gratitude for himself, but for the branch of medicine of which he is so important a member that the King should have selected him as a recipient for honour.

